# Comparison of different approaches to estimate bark volume of industrial wood at disc and log scale

**DOI:** 10.1038/s41598-021-95188-z

**Published:** 2021-08-02

**Authors:** Ferréol Berendt, Felipe de Miguel-Diez, Evelyn Wallor, Lubomir Blasko, Tobias Cremer

**Affiliations:** 1 Department of Forest Utilization and Timber Markets, Faculty of Forest and Environment, Eberswalde University for Sustainable Development, 16225 Eberswalde, Germany; 2 Chair of Forest Operations, Faculty of Environment and Natural Resources, University of Freiburg, 79085 Freiburg, Germany; 3 Department of Environmental Data Analysis and Programming, Faculty of Forest and Environment, Eberswalde University for Sustainable Development, 16225 Eberswalde, Germany; 4 Department of Forest Harvesting, Logistics and Ameliorations, Technical University Zvolen, 960 01 Zvolen, Slovakia

**Keywords:** Environmental sciences, Physics

## Abstract

Within the wood supply chain, the measurement of roundwood plays a key role due to its high economic impact. While wood industry mainly processes the solid wood, the bark mostly remains as an industrial by-product. In Central Europe, it is common that the wood is sold over bark but that the price is calculated on a timber volume under bark. However, logs are often measured as stacks and, thus, the volume includes not only the solid wood content but also the bark portion. Mostly, the deduction factors used to estimate the solid wood content are based on bark thickness. The aim of this study was to compare the estimation of bark volume from scaling formulae with the real bark volume, obtained by xylometric technique. Moreover, the measurements were performed using logs under practice conditions and using discs under laboratory conditions. The mean bark volume was 6.9 dm^3^ and 26.4 cm^3^ for the Norway spruce logs and the Scots pine discs respectively. Whereas the results showed good performances regarding the root mean square error, the coefficient of determination (R^2^) and the mean absolute error for the volume estimation of the total volume of discs and logs (over bark), the performances were much lower for the bark volume estimations only.

## Introduction

In order to tackle climate change and mitigate global warming, forests play an important role: (1) through carbon sequestration and (2) through reducing carbon emissions by providing both wood products and bioenergy to store carbon and as a substitute for other emission-intensive products and fossil fuel energy ^46^. Nevertheless, forests are affected by climate change, and, thus, “policies and plans must account for the trade-offs between forests’ capacity to store carbon, adapt to climate change and yield wood products and other ecosystem services” ^47^. To make economic processes and products more sustainable, member states of the European Union have adopted strategies in support of a renewable resource-based bioeconomy^1^. Especially the so-called wood-based bioeconomy is mainly driven by wood from forests as round timber, pulpwood and forest residues^2^. More recently, besides timber, new biomaterials made from wood and bark are getting more and more important, with steadily growing market segments. Bark is very promising as its unique chemical composition allows to get many different products (e.g. tannin and betulin) from which various materials can be produced^3,4^. For different species and diameters, the share of bark is very variable and ranges from as little as 4% to as much as 30% of the total over bark volume and weight^5^. In Central Europe raw timber is usually sold with bark, though customers pay for the volume of merchantable timber only, which is estimated under bark^6^. Therefore, “due to its substantial economic impact, the accurate prediction of the tree bark volume is of utmost importance”^7^.

Most of the research on bark proportions is focussed on the bark thickness because bark factors and bark functions are used to predict the diameter under bark (d_u.b._) from the diameter over bark (d_o.b._), in order to estimate the merchantable timber volume under bark (V_u.b._). To establish such bark functions, bark thickness is measured either using a bark gauge^8–10^ or by the difference of measurements between d_o.b._ and d_u.b._^11–13^. From log diameter and bark thickness, the volume of the logs over bark (V_o.b._) and under bark are estimated using taper functions or scaling formulae^6,8,9,13–17^. However, such log volume estimates result in bias and volume estimation errors^18–20^. In opposite, real volumes can be obtained using a xylometer which is also known as the fundamental measurement of water displacement. The volume obtained by immersion of logs is seen as reference, real or true volume and is therefore often used to describe the accuracy of log volume estimates^18–21^. Bark volume are calculated as the ratio^13^ or as the difference of the estimated V_o.b._ and V_u.b_^8,22^. Nevertheless, to the best of our knowledge, no scientific study analysed the accuracy of those formulae to estimate bark volume using water displacement methods.

Therefore, the purpose of this study was to evaluate the performance of bark volume estimation through mathematical formulae against water displacement technique. A second purpose of this study was to compare measurements performed under laboratory conditions on wood discs against measurements from real world conditions on logs. While for logs, the most widely known sectional methods for volume estimations (Huber, Smalian and Newton)^23,24^ were used, volume of wood discs was determined through geometric formula. The accuracy of volume over bark, volume under bark and bark volume was evaluated statistically.

## Results

### Wood discs

**Figure 1:**
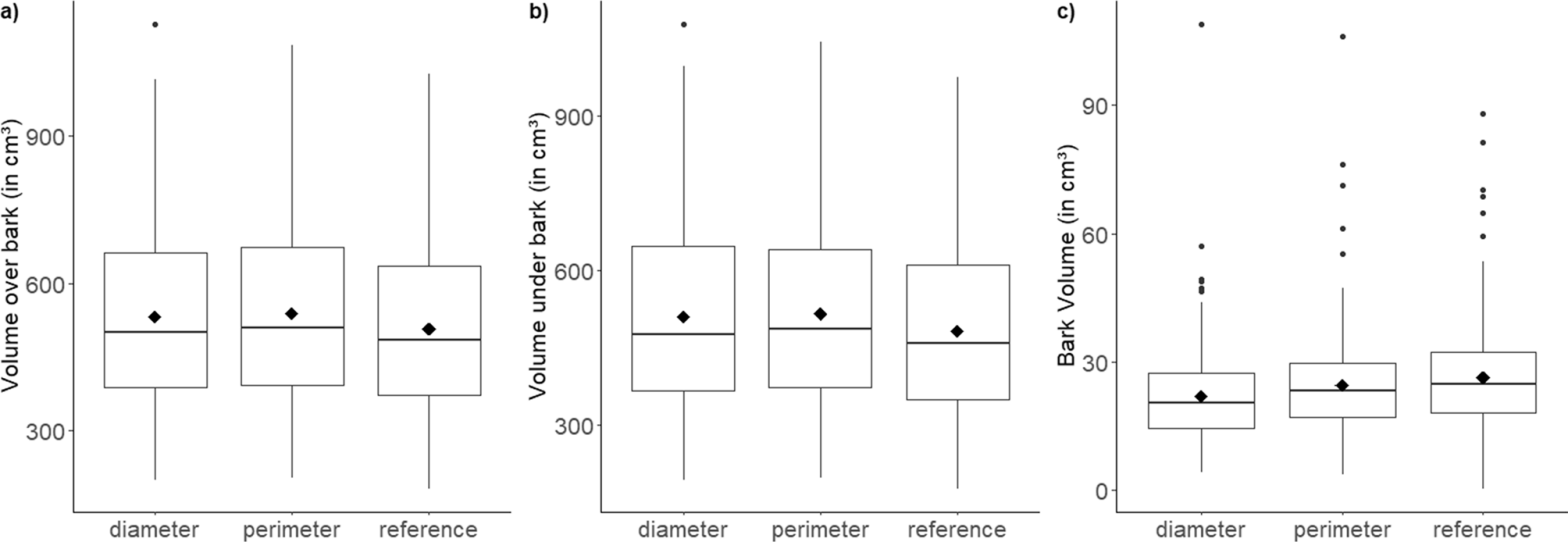
Boxplots of the (**a**) over bark volume, (**b**) under bark volume and (**c**) bark volume of wood discs for both diameter (Eq. 2) and perimeter (Eq. 3) volume estimation and for the reference volume.

Mean bark volume (as the difference of V_o.b._ and V_u.b._) was highest for the reference volume (26.4 cm^3^) followed by the perimeter equation (24.6 cm^3^) and diameter equation (22.1 cm^3^). Contrary, mean V_o.b._ and mean V_u.b._ was lowest for the reference compared to volume estimation by formulae (Fig. 1). The one-Way ANOVA showed that the means of V_o.b._ and V_u.b._ weren’t differing significantly from each other (*p* = 0.210 and *p* = 0.144, respectively) for the three independent variables, whereas the means of the different bark volumes differed significantly. According to the TukeyHSD test for the bark volume, only the two independent variables ‘reference’ and ‘diameter’ differed in a highly significant (*p* = 0.0015) way from one other. The deviation between the reference volume and both volume estimations ‘diameter’ and ‘perimeter’ is shown in Fig. 3.

The comparison of the disc’ volume (both V_o.b._ and V_u.b._) determined with perimeter or diameter and the reference volume showed a R^2^ above 0.98 in all cases while for the bark volume the R^2^ was below 0.40 for both equations (Table 1). When looking at RMSE and MAE the performances were better for V_o.b._ than for V_u.b._ and in both case for the volume estimation with diameter. For V_bark_, RMSE was 11.83 and 13.10 for the volume estimation with diameter and perimeter, respectively. On the contrary, MAE of V_bark_ was lower for the volume estimation with perimeter (8.65) compared to the diameter (8.90). When expressed as percentage of the reference volume, RMSE of V_bark_ reached approximately 50%, while for V_o.b._ and V_u.b._ it was below 8%. Similar results were found for MAE when calculated as a ratio to the reference volume with values around 5% for V_o.b._ and V_u.b._, while being around 36% for V_bark_.

**Table 1:** RMSE (root mean square error), R^2^ (coefficient of determination) and MAE (mean absolute error) of volume estimations with diameter (Eq. 2) and perimeter (Eq. 3) as input for the calculation to reference volume obtained by xylometry for the volume over bark (V_o.b._), volume under bark (V_u.b._) and bark volume (V_bark_) of wood discs.

	RMSE	R^2^	MAE
**V**_**o.b**_			
Equation 2 (diameter)	34.99	0.98	27.60
Equation 3 (perimeter)	39.31	0.99	33.09
**V**_**u.b**_			
Equation 2 (diameter)	37.07	0.99	30.10
Equation 3 (perimeter)	40.62	0.99	34.47
**V**_**bark**_			
Equation 2 (diameter)	11.83	0.37	8.90
Equation 3 (perimeter)	13.10	0.22	8.65

### Logs

**Figure 2:**
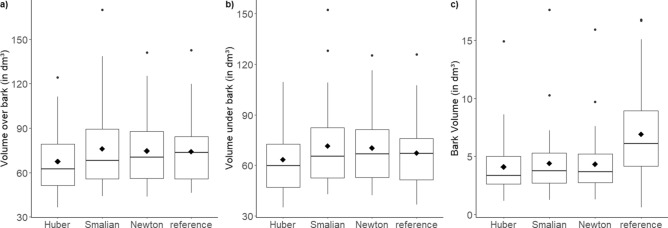
Boxplots of the (**a**) over bark volume, (**b**) under bark volume and (**c**) bark volume for the reference volume and volume estimated by Huber (Eq. 4), Smalian (Eq. 5) and Newton (Eq. 6) scaling formulas for spruce logs.

**Figure 3:**
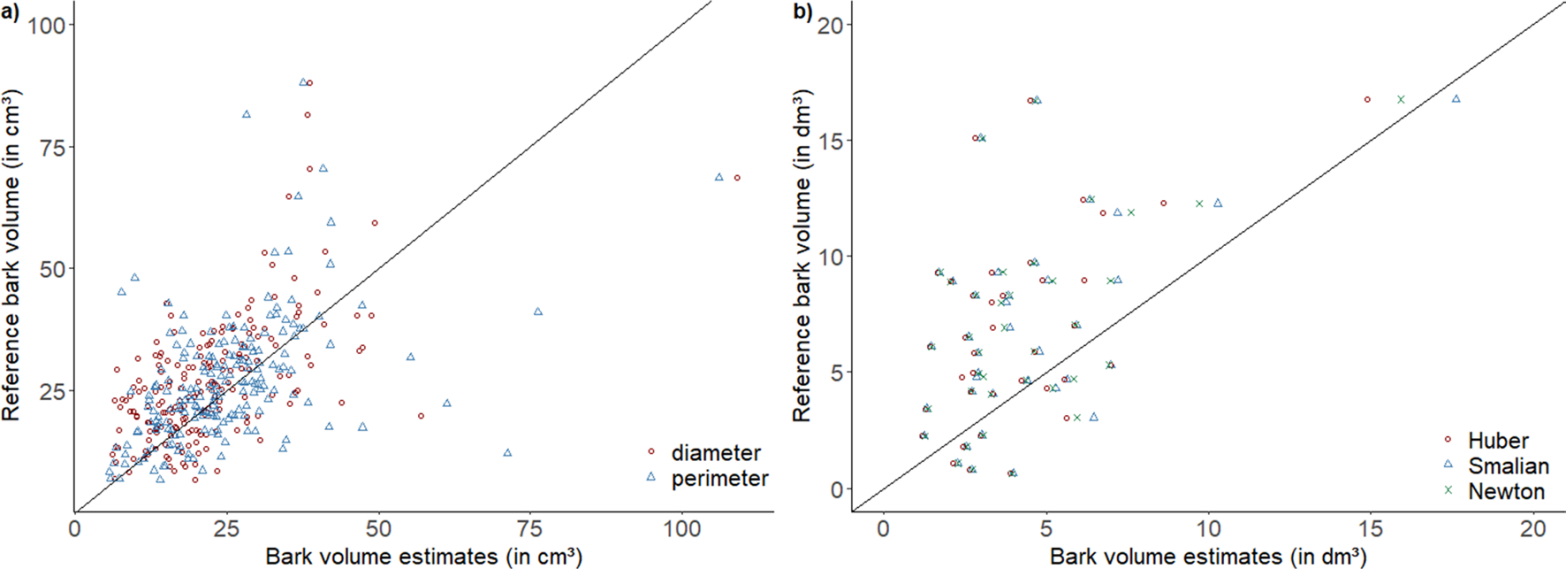
Scatterplots showing the reference bark volume (immersion) against bark volume estimations for (**a**) wood discs using the diameter (Eq. 2) and perimeter (Eq. 3) and for (**b**) logs using the scaling formulas Huber (Eq. 4),Smalian (Eq. 5) and Newton (Eq. 6).

The differences between the means of V_o.b._ estimated with scaling formulas compared to the reference volume were 9.0, 2.4 and 0.6% for Huber’s, Smalian’s and Newton’s formula, respectively. However, for both V_o.b._ and V_u.b._, no significant difference was observed according to the one-way ANOVA test (*p* = 0.398 and *p* = 0.365, respectively). The statistical analyses of the bark volumes of the logs showed that the reference volume showed highly significant differences to the other three independent variables ‘Huber’ (*p* = 0.0013), ‘Smalian’ (*p* = 0.0053) and ‘Newton’ (*p* = 0.0038). In contrast, the means of bark volume estimates (‘Huber’, ‘Smalian’ and ‘Newton’) did not differ significantly from each other. The mean reference bark volume was 6.90 ± 4.20 dm^3^, while the bark volumes estimated with Huber, Smalian and Newton scaling formulas were lower with 4.1 ± 2.5, 4.4 ± 3.0 and 4.3 ± 2.7, respectively (Fig. 2). This underestimation is also shown in Fig. 3 where the reference bark volume is plotted against the estimated bark volumes.

For V_o.b._ V_u.b._ the Newton’s scaling formula perform best (lowest RMSE and MAE and highest R^2^) followed by Smalian and Huber (Table 2). For example, the MAE of V_o.b._ was 7.39, 4.83 and 3.36 for Huber, Smalian and Newton scaling formulae, respectively. Looking at V_bark_, the MAE was of similar amplitude with values between 3.35 and 3.52, notwithstanding that the bark volume represented only 10% of V_o.b._. This is in line with lower performances with V_bark_ when looking at R^2^ compared to the R^2^ obtained when considering V_o.b._ and V_u.b._ as R^2^ was below 0.30. This was also found when comparing the ratio of RMSE of the Newton scaling formula to the reference volume: while the ratio was 6.38 for V_o.b._, it was around ten times higher for V_bark_ (63.33).

**Table 2:** RMSE (root mean square error), R^2^ (coefficient of determination) and MAE (mean absolute error) of volume estimations with the scaling formulas Huber (Eq. 4), Smalian (Eq. 5) and Newton (Eq. 6) as input for the calculation to reference volume obtained by xylometry for the volume over bark (V_o.b._), volume under bark (V_u.b._) and bark volume (V_bark_) of spruce logs.

	RMSE	R^2^	MAE
**V**_**o.b**_			
Huber (Eq. 4)	10.10	0.87	7.39
Smalian (Eq. 5)	7.34	0.95	4.83
Newton (Eq. 6)	4.73	0.96	3.36
**V**_**u.b**_			
Huber (Eq. 4)	8.23	0.87	5.67
Smalian (Eq. 5)	7.75	0.94	5.72
Newton (Eq. 6)	5.61	0.95	4.45
**V**_**bark**_			
Huber (Eq. 4)	4.53	0.25	3.52
Smalian (Eq. 5)	4.38	0.26	3.50
Newton (Eq. 6)	4.37	0.26	3.35

## Discussion and conclusion

The measurement of round wood is probably one of the most important elements of the wood supply chain^25^. The main reason of the importance of wood measurement is the economic impact, as wood cubage is one of the essential elements of wood value^20,26^. Wood industry mostly processes only the solid wood content without bark. Bark therefore often remains an unutilized industrial by-product which is used to produce energy only. However, the extraction of valuable compounds offers interesting uses for bark, but its potential for new value-added products is not yet fully exploited^27,28^. This is why, the interest in bark factors, which allow to estimate the proportion of bark of logs increased^29^. Such equations or models are based on bark thickness measurements. A common method used to measure the bark thickness, which is further used to develop and validate equations and models, is the bark gauge^8,9,29–32^. Such manual measurement result in over- or under-estimation of the real bark thickness as mentioned by several authors^9,14,31^. However, even if the choice of equation used in determining volume of bark is a source of error^20^, bark factors with a focus on bark thickness are well studied and the performances of several equations for different tree species show good results^10,11,16,30,33^. Such bark thickness factors are for example used when harvesters estimate under-bark volumes from over-bark measurements^6,34^. In this study we analyzed the accuracy of bark volume estimation on discs under laboratory conditions and on logs under real world conditions. Equations and scaling formulae to determine both V_o.b._ and V_u.b._ showed high performances at disc and log scale. Interestingly, the performances for bark volume estimations, as the difference of V_o.b._ and V_u.b._, were remarkably lower. According to the high performances for disc or log volume estimations and the low performance for V_bark_ estimations, when bark volumes are calculated as the difference between over-bark and under-bark volume, no conclusions on the accuracy of V_bark_ obtained by estimations or by immersion could be pronounced.

Considering that the bark of the pine discs was very thin with a double bark thickness of approximately 3.0 mm^35^ and a bark volume between 5 and 6%, the precision of the measurement is of high importance. The higher precision in the measurement equipment, caliper and weighing scale, may be an explanation why the relative performances were better for the discs than for the logs. For the discs and for the logs, bark volume estimated by equations underestimated the reference bark volume with lowest bias of 7.4% for the discs and of 36.1% for the logs in relation to the reference mean bark volume. The lower relative performances for the logs compared to the disc’s bark volumes were also observed for the other performance criterions. Nevertheless, bark volume of the logs is in accordance with other studies. Using the reference values, bark proportion of the spruce logs reached 9.3% compared to 11.5% for mean diameters of 22.5 cm^16^ and 10.8% at 30% relative height^22^. Thus, we assume that the source of error is not primary caused by the bark thickness measurement but by the choice of the equations.

While the high quality logs are mostly sold as stem wood, lower qualities logs are often designated for industrial wood purposes such as pulp, paper and particle board but also for energy. In Germany, one by one measurement of logs is widely used at the infeed of sawmills through opto-electronic devices after the debarking of the stem wood, while industrial wood is often measured as stacked wood. Measuring stacked wood includes the application of so-called reduction-factors to consider the volume of air space etc. in the stack^36^. Such reduction factors need to be reviewed regularly as the bark thickness became smaller in more recent assessments^32^, which underlines the high importance of accurate and regular measurement of bark proportions. Scaling formulas for estimating log volumes over and under bark are commonly used. The results showed that volume estimation with Newton’s formula differed to a lesser extent from the reference volume determined by xylometric method. However, its application is limited to research studies or experimental techniques since it is not common to measure the diameters at three points when commercializing round wood. The same inconvenience can be found using the Huber’s formula when purchasing industry wood^37^. As industrial wood assortments are usually sold in stacks, the measurement of the midpoint diameter is mostly not possible^38^. Due to this fact, the Smalian’s formula is more frequently applied.

As stated by several authors, the Newton’s formula is the most accurate equation to estimate the volume^39,40^, which is in line with the results of this study. The Smalian’s formula will overestimate the volume of a neiloid log, and in contrast, the Huber’s formula will underestimate it, even though its accuracy is better than the Smalian’s equation^39,40^. These statements are generally endorsed by the results of this study, except for the accuracy of the Huber’s formula, which was lower than the Smalian’s formula. While the results derived from applying the Smalian’s formula overestimated the volume, using Huber’s formula resulted in a volume underestimation compared to the reference volume. This fact is due to the specific features, especially the taper of the selected tree species: Norway spruce is characterized by an appreciable taper degree, even though the logs are short as in this exemplary case. Such deviations in volume estimations are enhanced further by butt-cut logs^37,40^. Other formulas to estimate log volumes exist but do not have a major role in forest mensuration or the application of such formulas is reduced to some regions or countries^37,41^. The visual interpretation of Fig. 3 showed that the bark volume estimates with the three scaling formulas are highly similar and, thus, strengthens the statistical analysis.

As with the water displacement technique includes all gaps and cracks it is seen as the real volume. It is probably one of the reasons why volume estimations underestimated V_bark_ compared to the reference volume. Moreover, the harvester’s feed rollers and/or delimbing knives causes damage to the wood and bark^35,42,43^, which may enhance the V_bark_ underestimation by formulas. According to Fig. 3, the trend of underestimation of bark volume when using formulas is more pronounced for the logs than for the discs. For the discs the underestimation seems to be more systematic for the ‘diameter’ than for the ‘perimeter’ estimation. The displaced water was quantified with weighing scale which may also lead to some error. Therefore, new methods or techniques should be compared to the water displacement technique. New technologies, such as computer tomography, could be used to quantify bark volume with greatest accuracy. This technology was already applied to determine the bias of bark thickness measurement with bark gauge^31^. Nevertheless, such methods are linked with high investments. On short length (i.e. discs), the diameter variation is not as pronounced as on logs. Moreover, it is recommended to reduce, if possible, the length of the logs when estimating their volume in order to reduce the error margin which is affected by the logs taper^37^. Therefore, the bark volume measurement on wood disc with water displacement technique was found to be a suitable method which is furthermore less time intensive than the xylometric measurement of logs. Moreover, the precision increased when analyzes, such as bark thickness measurements and immersion, were performed under laboratory conditions with more sensitive materials.

## Methods

The samples were derived from the forestry districts of Melchow (52°48′22″ N; 13°42′13″ E) and Kahlenberg (52°52′43″ N, 13°53′17″ E), located in the federal state of Brandenburg, Northeast Germany.

Volume over bark (V_o.b._) and Volume under bark (V_u.b_)_._ of 250 wood discs and 37 logs were determined with water immersion technique. As bark losses typically occur on the logs way from forest to mill and depending on log handling practices and season^5^, the degree of bark damage was evaluated for each log and disc. Measurements of missing bark were carried out using measurement tape and calculating the ratio of missing bark to the perimeter. For logs every 25 cm the measurement was repeated ten times and the average value was estimated. For discs two measurements were considered as sufficient.

### Wood discs

250 wood discs were sawn from 50 different Scots pine (*Pinus sylvestris*, L.) logs felled between January and April 2020. Mean diameter over bark (d_o.b._) of the discs was 13.0 ± 2.2 cm and mean disc thickness (h) was 3.9 ± 0.6 cm. Dimensions were measured with precision calliper (diameter), measuring tape (perimeter) and a lab xylometer (volume), adapted to the disc’s volume. Diameter over bark (d_o.b._) and diameter under bark (d_u.b._) were both measured twice perpendicularly. More detailed information about the measurement methods are described by Berendt et al.^35^. The reference V_o.b._ and V_u.b._ of the wood discs was obtained by dividing the mass of displaced water (m_water_) by water density (ρ_water_):

1 $$V=\frac{{m}_{water}}{{\rho }_{water}}$$

All measurements were executed under laboratory conditions and a value of 0.9985 was applied for ρ_water_. The discs were immersed into a 30 cm diameter and 10 cm deep water basin equipped with an overflow device. During the immersion the samples were in green condition and the moisture content (MC) of all analyzed samples was above fiber saturation point (MC > 30%). Moreover, the immersing time of the wood was merely a few minutes. Thus, the penetration of water into the wood and increase in volume are negligible.

Besides the reference volume determination with xylometer, volume of the discs was estimated with the geometric formula of a cylinder. Volume was estimated with (1) the diameter (d) measured with precision caliper (Eq. 2) and (2) the perimeter (P) measured with measuring tape (Eq. 3):

2 $$V=\uppi \frac{{d}^{2}}{4}h$$

3 $$V=\frac{P}{4\uppi }h$$

Bark volume (V_bark_) was calculated as the difference between V_o.b._ and V_u.b._ for both the predicted and the reference V_bark_.

### Wood logs

The bark volume of 37 spruce logs (*Picea abies*, H. Karst) was quantified. The analysed logs were 2.53 ± 0.02 m long and had mean diameter over bark at mid-length of 19.08 ± 2.65 cm. Reference bark volume was defined as the difference of V_o.b._ and V_u.b._, both measured with water immersion technique. Bark was peeled with a barking iron.

Log volumes were also estimated using the most widely used sectional methods for volume estimation based on tree stem geometry, more specifically on cross-sectional areas^6^: Huber (Eq. 4), Smalian (Eq. 5) and Newton (Eq. 6).

4 $$V=M*L$$

5 $$V=\frac{\mathrm{B}+\mathrm{S}}{2}*L$$

6 $$V=\frac{\mathrm{B}+4\mathrm{M}+\mathrm{S}}{6}*L$$

where V = volume, B = cross-sectional area at large end of log (m^2^), M = cross-sectional area at mid-length of log (m^2^), S = cross-sectional area at small end of log (m^2^), L = log length (m). The cross-sectional areas were calculated with two perpendicular diameter measurements done with calliper. The bark thickness, which was determined with a mm-precision, was subtracted from the mean diameters to estimate V_u.b._ with the same scaling formula. Finally, V_bark_ was calculated as the difference of V_o.b._ and V_u.b._.

### Statistics

With statistical analysis the performance of the different scaling formulas compared to the reference, was evaluated by root mean square error (RMSE) (Eq. 7), coefficient of determination (R^2^) (Eq. 8) and mean absolute error (MAE) (Eq. 9). RMSE^44^ is a common indicator for calibration. Due to its quadratic nature RMSE is very sensitive to outliers. In contrast, MAE averages the absolute, unaltered values and is thus more robust against unequally distributed error populations^45^. Calculation of RMSE and MAE was done in R-3.6.2 with the Metrics package, whereas R^2^ was determined with the function ‘summary’ of a linear model (lm). For RMSE and MAE, the model that had the lowest value perform best while the model with a value closest to one was best for R^2^.

7 $$RMSE = \sqrt {\mathop \sum \limits_{i = 1}^{n} \frac{{\left( {V - P} \right)^{2} }}{n}}$$

8 $$R^{2} = 1 - \left[ {\mathop \sum \limits_{i = 1}^{n} \frac{{\left( {V - P} \right)^{2} }}{{\left( {V - \overline{V}} \right)^{2} }}} \right]$$

9 $$MAE = \mathop \sum \limits_{i = 1}^{n} \frac{{\left| {V - P} \right|}}{n}$$

where $V$ = reference volume, $\hat{P}$ = predicted volume, $\overline{V}$ = reference mean volume and $n$ = number of observations.

Moreover, a one-way ANOVA was done to determine whether the means from the reference volumes and the different volume estimates differ significantly. A TukeyHSD was performed as post-hoc test in order to identify which groups differ from each other. The three independent variables for the wood disc analysis were (1) reference volume (‘reference’) (2) volume estimate with Eq. 2 (‘diameter’) and (3) volume estimate with Eq. 3 (‘perimeter’). For the logs, four independent variables were considered: (1) reference volume (‘reference’), (2) volume estimate with Huber scaling formula (‘Huber’), volume estimate with Smalian scaling formula (‘Smalian’) and volume estimate with Newton scaling formula (‘Newton’). As the significance level α was defined with 0.05, a *p *value < 0.05 provided a statistically significant result in the ANOVA and TukeyHSD test. A *p *value < 0.01 was considered as highly significant.

### Plant material

Permission to collect *Pinus sylvestris* and *Picea abies* were obtained by the forest owner (Landeswaldoberförsterei Chorin). The handling of the wood samples were carried out in accordance with relevant guidelines and regulations.
